# Association between quantitative flow ratio and clinical outcomes in multivessel disease STEMI patients with diabetes mellitus

**DOI:** 10.1371/journal.pone.0313892

**Published:** 2024-12-05

**Authors:** Huimin Xian, Xing Luo, Yanzong Liu, Bingchen Guo, JianJun Wu, Fan Yang, Yiyuan Guo, Ruoxi Zhang

**Affiliations:** 1 Department of Cardiology, The 2nd Affiliated Hospital of Harbin Medical University, Harbin, China; 2 Department of Cardiology, Xiamen Soong Hospital, Xiamen, China; 3 Department of Cardiology, The 1st Affiliated Hospital of Harbin Medical University, Harbin, China; 4 Department of Ophthalmology, The 1st Affiliated Hospital of Harbin Medical University, Harbin, China; 5 Department of Cardiology, Harbin Yinghua Hospital, Harbin, China; University of Massachusetts Medical School, UNITED STATES OF AMERICA

## Abstract

**Background:**

Among patients with multivessel disease and ST-elevation myocardial infarction (MVD-STEMI), complete revascularization (CR) has been shown with improved outcomes. However, it is controversial whether diabetes mellitus (DM) status affects the outcomes. Quantitative flow ratio (QFR), as a newer non-invasive tool for identifying functional coronary stenosis and determining the presence of functional CR (FCR), may open up a new perspective for studying the above issues. The aim of this retrospective study was to investigate an association between QFR-based FCR and clinical outcomes in MVD-STEMI patients under DM status.

**Methods:**

A total of 623 patients were included in the final analysis. The patients were divided into nonDM cohort and DM cohort. Within each cohort, patients were further stratified into functional CR (FCR) layer and functional incomplete revascularization (FIR) layer based on QFR assessment. The primary outcomes were 3-year major adverse cardiovascular events (MACEs), encompassing cardiac death, ischemia-driven revascularization (target vessel and non-target vessel), rehospitalization due to unstable angina pectoris, and non-fatal myocardial infarction.

**Results:**

The incidence of MACEs was significantly lower in the FCR layer than in the FIR layer (12.6% vs 24.0%, log-rank *P<*0.001). In the nonDM cohort, the incidence of MACEs was also lower in the FCR layer than in the FIR layer (9.8% vs 18.5%, log-rank *P* = 0.032). Similar situations occurred in the DM cohort (16.1% vs 27.9%, log-rank *P* = 0.017). In addition, the multivariate Cox analysis showed that rSS_QFR_ (QFR-derived residual SYNTAX score) was significantly associated with the increased risk of MACEs in the nonDM cohort (HR (95% CI) = 1.18 (1.10–1.26), *P*<0.001) and DM cohort (HR (95% CI) = 1.13 (1.09–1.18), *P*<0.001). ROC analysis showed adding rSS_QFR_ into the model of clinical risk factors yielded a significant improvement in prediction of MACEs, especially in the DM cohort (AUC (95% CI) = 0.747 (0.675–0.819), *P* = 0.001) than in the nonDM cohort (AUC (95% CI) = 0.697 (0.602–0.791), *P* = 0.033). Furthermore, additional multivariate Cox analysis showed that rSS_QFR_ was associated with the increased risk of MACEs in patients with moderate lesions (DS of 50%-89%) after procedure (HR (95% CI) = 1.16 (0.11–1.22), *P*<0.001).

**Conclusions:**

In patients with MVD-STEMI, the incidence of MACEs was lower in FCR than in FIR, and the decrease was particularly significant in the DM cohort. The association between QFR-derived rSS_QFR_ and MACEs was independent of baseline characteristic differences, and rSS_QFR_ provided higher prognostic predictive ability in DM cohort than in nonDM cohort. Additionally, QFR had the additional utility of identifying moderate residual lesions that require revascularization.

## Introduction

About 40%-50% of patients presenting with ST-elevation myocardial infarction (STEMI) have multivessel disease (MVD). Compared with patients with single-vessel disease, patients with MVD-STEMI have higher mortality rates and poorer prognosis [1]. Percutaneous coronary intervention (PCI) is a crucial treatment modality for coronary disease patients [1]. Complete revascularization (CR) strategy plays a significant role in managing MVD-STEMI patients as it enables comprehensive revascularization of all stenoses, thereby exerting a direct and effective impact on improving outcomes [1].

Diabetes mellitus (DM) is an important risk factor for coronary artery. It is associated with a more complex coronary abnormalities including diffuse atherosclerotic plaques with calcification, negative remodeling, and the frequent presence of microvascular disease [2–4]. Nevertheless, there remains controversy regarding whether MVD-STEMI patients combined with DM can still benefit from CR strategy [5, 6].

Quantitative Flow Ratio (QFR) stands as a newer functional assessment tool for evaluating coronary stenosis and was an important alternative to fractional flow reserve (FFR) [7, 8]. Notably, QFR eliminates numerous disadvantages associated with FFR, such as the requirement for additional invasive pressure wires or hyperemia induction [7, 8]. In the context of PCI, studies have shown that QFR-guided lesion selection strategies can significantly enhance patient prognosis [9]. In fact, compared to angiography-guided CR strategies, QFR-guided functional CR (FCR) has been found to reduce the 1-year rate of major adverse cardiac events (MACEs) in patients with coronary artery disease (CAD) [9]. Despite these advancements, there is still a lack of reports exploring the application of QFR in MVD-STEMI patients combined with DM.

In this study, we aim to retrospectively investigate the association between QFR-based FCR and clinical outcomes in MVD-STEMI patients with/without DM.

## Methods

### Study population and design

The present report was a single-center, retrospective, observational study. A total of 724 consecutive patients were included at the 2nd Affiliated Hospital of Harbin Medical University from January 2017 to December 2018. The inclusion criteria: adult MVD-STEMI patients with or without DM underwent primary PCI based on standard angiography guidance; sufficient coronary angiography images. The exclusion criteria: general exclusion criteria: left main disease, previous coronary artery bypass grafting (CABG) history, hemodialysis, cardiogenic shock, glucose data missing, CABG in follow-up; angiographic exclusion criteria: ostial lesions, severe overlap, lack of 2 appropriate projections.

Each MVD-STEMI patients had one infarct-related artery (IRA) and at least one non-IRA. For IRA, all patients underwent primary PCI treatment, while for non-IRA, patients selectively underwent primary PCI, staged PCI, or no treatment, depending on the severity of the stenosis and the decision of interventional cardiologist and the patient themselves. Angiography images were collected and the postoperative QFR value after the last PCI was measured. For the determination of last PCI, if the patient underwent staged PCI, the last PCI was considered as staged PCI. If the patient had not undergone staged PCI, the last PCI was considered as primary PCI. Afterwards, the rSS_QFR_ (residual Synergy Between Percutaneous Coronary Intervention With Taxus and Cardiac Surgery (SYNTAX) score derived from QFR) score was further calculated based on the postoperative QFR value after the last PCI.

**Fig 1:**
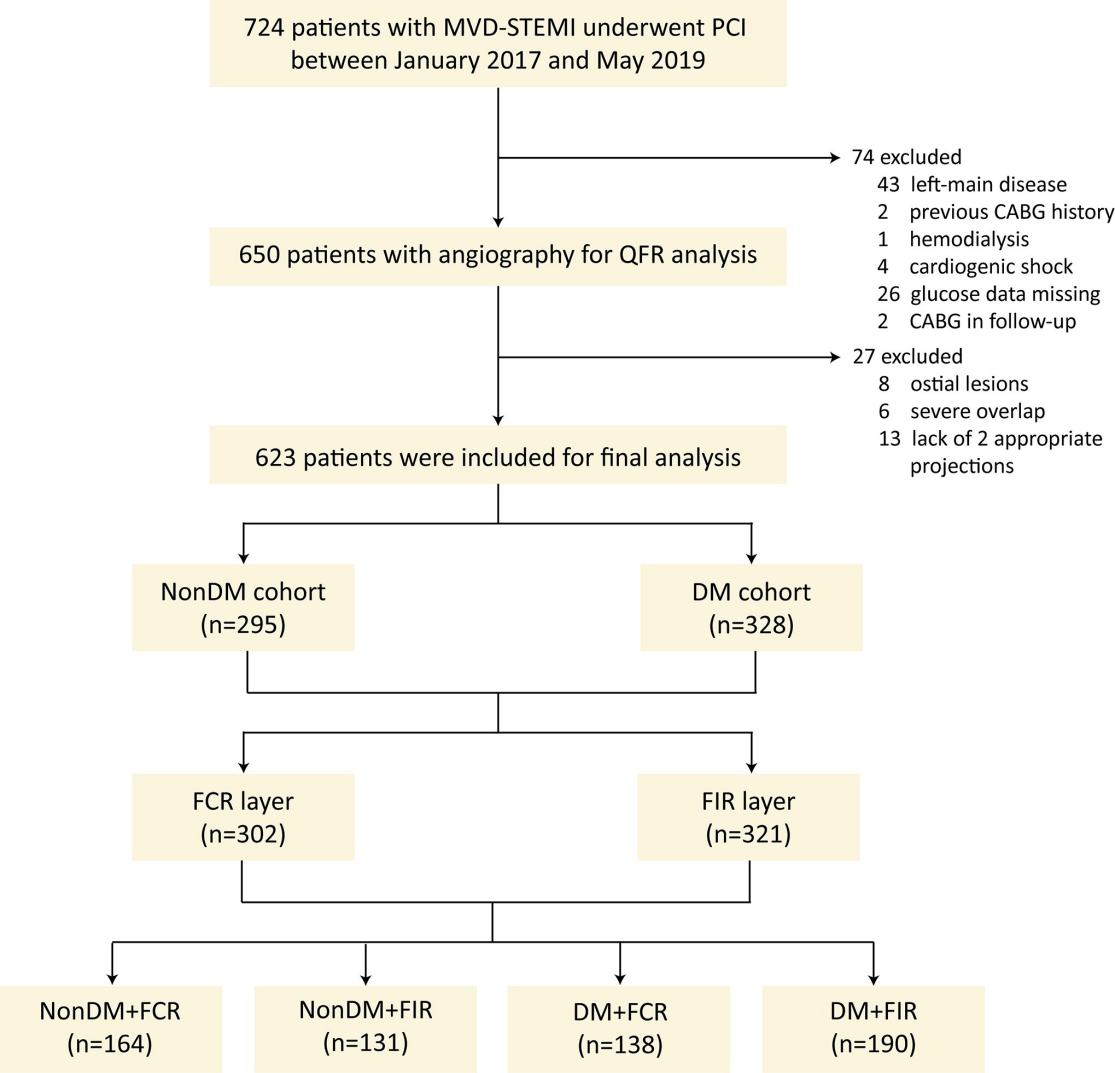
Study flowchart.

The patients were categorized based on the diagnostic criteria of DM and rSS_QFR_ score. According to the diagnostic criteria of DM, patients were divided into nonDM cohort (n = 295) and DM cohort (n = 328). In each cohort, patients were further divided into FCR layer (n = 302) and functional IR (FIR) layer (n = 321) based on rSS_QFR_. The rSS_QFR_ served as a metric to assess the degree of functional vascular stenosis by QFR techniques [10]. A score of 0 indicated the absence of residual functional stenosis postoperatively, thus qualifying as FCR [11]. Conversely, a score greater than 0 implied the presence of residual functional stenosis postoperatively, categorized as FIR [11]. As a result, patients were divided into four groups: nonDM+FCR (n = 164, rSS_QFR_ = 0), nonDM+FIR (n = 131, rSS_QFR_>0), DM+FCR (n = 138, rSS_QFR_ = 0), and DM+FIR (n = 190, rSS_QFR_>0). This stratification was graphically represented in the study flowchart (**Fig 1**). Specific definitions see S1 File.

### Study procedures

All patients received loading doses of aspirin (300 mg), clopidogrel (300 mg) or ticagrelor (180 mg) before primary PCI. The IRA were ascertained by evaluation of electrocardiographic changes, echocardiographic results and angiographic findings. Primary PCI was in compliance with the current guidelines and the operators’ routine practice. After the procedure, aspirin (100 mg/day) and clopidogrel (75 mg/day) or ticagrelor (180 mg/day) were prescribed at the same time daily. Staged PCI was performed no later than 30 days after primary PCI.

### Ethical approval

This observational study was conducted in compliance with the Declaration of Helsinki. The study was reviewed and approved by the ethics committee of 2nd Affiliated Hospital of Harbin Medical University (sydwgzr2020-128). All patients provided written informed consent for potential future investigations. The authors did not have access to any information that could identify individual participants during or after data collection.

### Off-line QFR assessment

The principle of QFR relies on angiography-based three-dimensional coronary reconstruction and frame counting techniques. Following the commencement of this study, QFR analysis was conducted offline by two independent, certified operators utilizing the AngioPlus system (Pulse Medical Imaging Technology, Shanghai, China). This measurement commenced at the ostium of the index vessel and extended to a distal anatomic landmark visible in both projections [8]. QFR analysis was performed preoperative and postoperative on all three vessels, inclusive of major side branches (such as the obtuse marginal branch, intermediate branch, and diagonal branch) with a vessel diameter of ≥1.5 mm. Subsequently, vessels with a conventionally defined cut-off QFR value of ≤0.80 were considered for further SYNTAX score calculations.

### Calculation of SYNTAX score

The extent of coronary obstruction can be accurately quantified and digitized using the SYNTAX score (SS) standard by comprehensively measuring indicators such as the number, location, and complexity of coronary artery. SS represents the baseline (preoperative) anatomical level by calculating preoperative coronary artery lesions before primary PCI [12]. The residual SS score (rSS) represents the postoperative anatomical level by calculating residual stenosis of coronary artery lesions after the last PCI. The preoperative QFR value before primary PCI and the postoperative QFR value after the last PCI were measured. If the QFR value was ≤0.80, it was determined there was functional coronary stenosis. Finally, SS_QFR_ and rSS_QFR_ were derived by calculating the SS of vessel with preoperational QFR value ≤0.80 before primary PCI and rSS of vessel with postoperative QFR value ≤0.80 after the last PCI, respectively [11, 13].

### Clinical outcomes

All patients were followed by phone or hospital visits at 6, 12, 18, 24, 30 and 36 months after discharge. MACEs were defined as a composite of cardiac death, ischemia-driven revascularization including target vessel revascularization (TVR) and non-target vessel revascularization (non-TVR), rehospitalization due to unstable angina pectoris (UAP), and non-fatal myocardial infarction (MI) [14, 15]. All endpoint events were adjudicated by an independent committee blinded to assignment.

### Statistical analysis

Statistical analysis were performed by SPSS, version 27.0, and Graphpad Prism, version 8.0. Categorical data was presented as counts (proportions) and compared by using the Pearson chi-square test or Fisher exact test depending on category cell size. Kolmogorov-Smirnov test was used to assess the normality of continuous data. Normally distributed variables were described as mean ± SD and compared by using the Student’s t-test, whereas non-normally distributed variables were described as median (interquartile range [IQR]) and compared by using the Mann-Whitney U test. Multivariable logistic regression analysis was used to identify the independent predictors of FIR. Variables with *P*<0.1 in the univariable analysis were taken into multivariate analysis. Time-to-event data was presented as cumulative incidence plots and compared by the log-rank test. Multivariable Cox regression was utilized to determine the relationship between rSS_QFR_ and MACEs and estimate between-group risks by hazard ratio (HR) with 95% confidence interval (CI). Adjustments were made for baseline characteristics, including age, male, smoking history, hypertension, dyslipidemia, chronic kidney diseases (CKD), previous MI, previous PCI, and non-IRA diameter stenosis (DS)≥90. Furthermore, Cox regression was also applied to analyse the MACEs components in different cohorts or layers. In cases where zero events were observed, both the experimental group and the control group added 0.5 events through data imputation techniques to facilitate subsequent analysis. Receiver-operating characteristic curve (ROC) analysis was employed to compare the recognition ability of MACEs between different models. The basic clinical risk factor model included age, male, smoking history, hypertension, dyslipidemia, CKD, previous MI, and previous PCI. A 2-tailed *P*<0.05 was considered to indicate statistical significance.

## Results

### Clinical characteristics

Among patients with MVD-STEMI, 47.4% patients had nonDM, of which 55.6% were FCR and 44.4% were FIR (**Fig 1**). The rest 52.6% patients had DM, of which 42.1% were FCR and 57.9% were FIR (**Fig 1**).

Compared to nonDM cohort, DM cohort had more current smoking, higher low-density lipoprotein cholesterol (LDL-C) and high-density lipoprotein cholesterol (HDL-C), higher peak cardiac troponin I (cTnI), higher hemoglobinA1c (HbA1c) and fasting blood glucose (FBG) (**S1 Table**). In the nonDM cohort, patients in FIR layer (nonDM+FIR) exhibited more hypertension than those in FCR layer (nonDM+FCR) (**Table 1**). In the DM cohort, patients in FIR layer (DM+FIR) had lower estimated glomerular filtration rate (eGFR) and more insulin use than those in FCR layer (DM+FCR) (**Table 1**).

**Table 1:** Clinical characteristics.

	NonDM cohort			DM cohort		
	NonDM+FCR (n = 164)	NonDM+FIR (n = 131)	*P* Value	DM+FCR (n = 138)	DM+FIR (n = 190)	*P* Value
Age, y	60.2 ± 11.0	60.9 ± 11.7	0.607	61.1 ± 10.6	61.5 ± 10.1	0.691
Male	106 (64.6)	84 (64.1)	0.927	89 (64.5)	120 (63.2)	0.804
Coronary risk factors						
Current smoking	95 (57.9)	74 (56.5)	0.804	68 (49.3)	90 (47.4)*	0.733
Hypertension	104 (63.4)	101 (77.1)	**0.011**	93 (67.4)	139 (73.2)	0.257
Dyslipidemia	129 (78.7)	93 (71.0)	0.130	98 (71.0)	139 (73.2)	0.669
CKD	41 (25.0)	34 (26.0)	0.852	33 (23.9)	49 (25.8)	0.698
Previous history						
Previous MI	1 (0.6)	3 (2.3)	0.215	5 (3.6)	5 (2.6)	0.571
Previous PCI	5 (3.0)	3 (2.3)	0.690	4 (2.9)	7 (3.7)	0.696
Laboratory data						
TC, mg/dl	4.8 ± 1.2	5.0 ± 1.1	0.244	5.0 ± 1.2	5.1 ± 1.2	0.494
TG, mg/dL	1.6 (1.1–1.9)	1.5 (1.1–2.2)	0.463	1.3 (0.9–1.9)	1.4 (0.9–2.3)	0.125
LDL-C, mg/dL	2.7 (2.3–3.4)	3.0 (2.3–3.5)	0.154	3.0 (2.5–3.7)^△^	3.2 (2.5–3.7)	0.651
HDL-C, mg/dL	1.2 ± 0.3	1.3 ± 0.3	0.365	1.3 (1.1–1.5)^△^	1.3 (1.1–1.5)	0.486
Peak cTnI, μg/L	57.4 (26.1–113.0)	64.3 (26.6–105.0)	0.785	72.6 (23.0–142.2)	89.5 (35.5–163.9)*	0.197
LVEF, %	61.0 (55.0–62.8)	61.0 (55.0–62.0)	0.908	61.0 (54.0–63.0)	59.0 (53.0–62.0)	0.218
eGFR, mL/min/1.73 m^2^	75.4 (60.1–94.2)	75.5 (58.2–99.8)	0.641	79.5 (60.9–94.9)	71.6 (60.0–87.8)*	**0.034**
HbA1c, %	5.6 (5.4–5.8)	5.6 (5.4–5.8)	0.618	6.4 (5.7–7.8)^△^	6.8 (5.7–8.0)*	0.179
FBG, mmol/L	5.3(4.9–6.2)	5.5 (4.8–6.1)	1.000	8.6 (7.4–11.2)^△^	9.1 (7.6–12.0)*	0.110
Diabetes management						
Diet or exercise	-	-	-	4 (2.9)	11 (25.8)	0.216
Oral agent	-	-	-	82 (59.4)	110 (57.9)	0.782
Injection	-	-	-	14 (10.1)	37 (19.5)	**0.021**
Untreated	-	-	-	42 (30.4)	49 (25.8)	0.354
Discharge medications						
Aspirin	169 (97.6)	131 (100.0)	0.072	136 (98.6)	185 (97.4)	0.465
P2Y12 inhibitors	161 (98.2)	131 (100.0)	0.120	138 (100.0)	188 (98.9)	0.227
Statins	162 (98.8)	127 (96.9)	0.268	137 (99.3)	189 (99.5)	0.820
Oral anticoagulant	19 (11.6)	13 (9.9)	0.648	14 (10.1)	23 (12.1)	0.580

Values are n (%), mean±SD, or median (interquartile range). Bold represented significance in the nonDM cohort or in the DM cohort. ^△^represents significance in the FCR layer. *represents significance in the FIR layer. *P*<0.05 was considered statistically significant.

### Procedural characteristics

Compared to nonDM cohort, DM cohort had higher SS, rSS, SS_QFR_ and rSS_QFR_, higher proportion of IRA with DS≥90, more non-IRA numbers, higher proportion of non-IRA with DS≥90 and QFR<0.8, and more number of stents (**S2 Table**). In the nonDM cohort, patients in FIR layer (nonDM+FIR) exhibited more 3-vessel diseases, higher SS, rSS, SS_QFR_ and rSS_QFR_, more non-IRA numbers, a higher proportion of non-IRA with DS≥90 and QFR<0.8, and more staged PCI for non-IRA than those in FCR layer (nonDM+FCR) (**Table 2**). In the DM cohort, patients in FIR layer (DM+FIR) were the same as those in nonDM cohort, except for patients with stent diameter and stent length greater than those in FCR layer (DM+FCR) (**Table 2**). The distribution of the rSS and rSS_QFR_ were presented in the violin plot (**S1 Fig**). The median time of staged PCI was 32 days in the nonDM cohort and 34 days in the DM cohort.

**Table 2:** Procedural characteristics.

	NonDM cohort			DM cohort		
	NonDM+FCR (n = 164)	NonDM+FIR (n = 131)	*P* Value	DM+FCR (n = 138)	DM+FIR (n = 190)	*P* Value
3-vessel diseases	75 (45.7%)	82 (62.6%)	**0.004**	65 (47.1%)	128 (67.4%)	**<0.001**
Syntax score						
Angiography-derived						
Pre-PCI (SS)	13.8 (11.0–19.0)	17.0 (13.0–21.5)	**<0.001**	13.0 (10.4–18.0)	18.0 (14.5–23.0)*	**<0.001**
Post-PCI (rSS)	5.0 (2.0–8.0)	8.0 (4.0–12.0)	**<0.001**	5.0 (2.0–7.0)	9.5 (6.0–13.0)*	**<0.001**
QFR-derived						
Pre-PCI (SS_QFR_)	6.0 (4.0–13.5)	11.0 (8.0–16.0)	**<0.001**	6.0 (5.0–13.5)	15.0 (9.9–20.5)*	**<0.001**
Post-PCI (rSS_QFR_)	0	2.5 (2.0–6.0)	**<0.001**	0	5.0 (2.0–9.0)*	**<0.001**
IRA						
Number	1	1	-	1	1	-
Initial TIMI flow grade ≤ 1	163 (99.4%)	129 (98.5%)	0.435	136 (98.6%)	182 (95.8%)	0.151
DS ≥ 90	164 (100%)	130 (99.2%)	0.262	136 (98.6%)	184 (96.8%)	0.322
QFR ≤ 0.8	148 (90.2%)	121 (92.4%)	0.523	126 (91.3%)	182 (95.8%)	0.094
Location						
LAD	65 (39.6%)	58 (44.3%)	0.422	59 (42.8%)	78 (41.1%)	0.758
LCX	18 (11.0%)	21 (16.0%)	0.203	18 (13.0%)	28 (14.7%)	0.663
RCA	81 (49.4%)	53 (40.5%)	0.126	62 (44.9%)	83 (43.7%)	0.823
Location of QFR ≤ 0.8						
LAD	61 (37.2%)	50 (38.2%)	0.864	54 (39.1%)	76 (40.0%)	0.874
LCX	18 (11.0%)	21 (16.0%)	0.203	15 (10.9%)	26 (13.7%)	0.447
RCA	69 (42.1%)	50 (38.2%)	0.497	57 (41.3%)	80 (42.1%)	0.885
Non-IRA						
Number						
Pre-PCI	1.0 (1.0–2.0)	2.0 (1.0–2.0)	**0.003**	1.0 (1.0–2.0)	2.0 (2.0–2.0)*	**<0.001**
Post-PCI	1.0 (1.0–2.0)	2.0 (1.0–2.0)	**0.012**	1.0 (1.0–2.0)	2.0 (1.0–2.0)*	**<0.001**
DS ≥ 90						
Pre-PCI	20 (12.2%)	58 (44.3%)	**<0.001**	20 (14.5%)	89 (46.8%)	**<0.001**
Post-PCI	11 (6.7%)	50 (38.2%)	**<0.001**	12 (8.7%)	82 (43.2%)	**<0.001**
QFR ≤ 0.8						
Pre-PCI	10 (6.1%)	119 (90.8%)	**<0.001**	11 (8.0%)	183 (96.3%)*	**<0.001**
Post-PCI	0	118 (90.1%)	**<0.001**	0	179 (94.2%)	**<0.001**
Location						
LAD	85 (51.8%)	71 (54.2%)	0.685	71 (51.4%)	107 (56.3%)	0.382
LCX	90 (54.9%)	86 (65.6%)	0.061	77 (55.8%)	133 (70.0%)	**0.008**
RCA	63 (38.4%)	56 (42.7%)	0.451	55 (39.9%)	101 (53.2%)	**0.017**
Location of QFR ≤ 0.8						
LAD	5 (3.0%)	48 (36.6%)	**<0.001**	6 (4.3%)	69 (36.3%)	**<0.001**
LCX	2 (1.2%)	65 (49.6%)	**<0.001**	5 (3.6%)	99 (52.1%)	**<0.001**
RCA	3 (1.8%)	36 (27.5%)	**<0.001**	1 (0.7%)	80 (42.1%)*	**<0.001**
Treatment						
Number of stents	1.0 (1.0–1.0)	1.0 (1.0–1.0)	0.132	1.0 (1.0–2.0)^△^	1.0 (1.0–2.0)	0.525
Stent diameter, mm	3.0 (2.9–3.5)	3.0 (2.8–3.5)	0.128	3.0 (2.8–3.5)	3.0 (2.8–3.2)	**0.020**
Stent length, mm	29.0 (23.0–36.0)	30.0 (24.0–38.0)	0.139	28.5 (23.0–39.3)	33.0 (24.0–48.0)	**0.040**
Thrombectomy	127 (77.4%)	96 (73.3%)	0.409	103 (74.6%)	131 (68.9%)	0.261
IRA PCI	100%	100%	-	100%	100%	-
Non-IRA PCI	16 (9.8%)	16 (12.2%)	0.500	13 (9.4%)	23 (12.1%)	0.442
Primary PCI	13 (7.9%)	4 (3.1%)	0.074	11 (8.0%)	7 (3.7%)	0.092
Staged PCI	3 (1.8%)	12 (9.2%)	**0.004**	2 (1.4%)	16 (8.4%)	**0.006**

Values are n (%), mean±SD, or median (interquartile range). Bold represented significance in the nonDM cohort or in the DM cohort. ^△^represents significance in the FCR layer. *represents significance in the FIR layer. *P*<0.05 was considered statistically significant.

Multivariate logistic regression analysis showed that SS_QFR_ and non-IRA DS≥90 were associated with the presence of FIR (**S2 Fig**).

### Clinical outcomes

**Fig 2:**
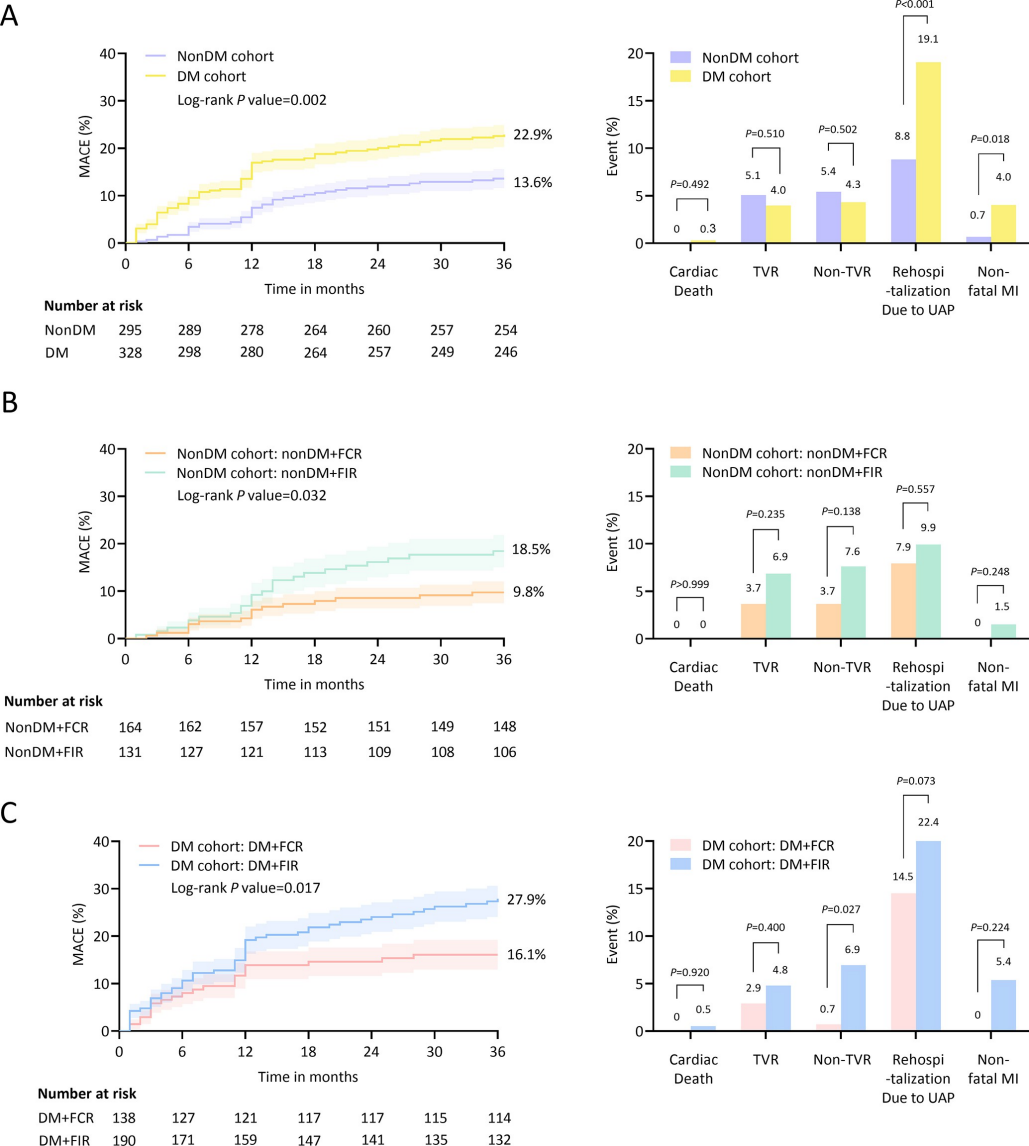
Cumulative incidence plots for 3-year clinical outcomes. MACEs plots and MACEs components histogram for comparison between nonDM and DM cohorts (**A**), comparison between FCR and FIR layers in the nonDM cohort (**B**), and comparison between FCR and FIR layers in the DM cohort (**C**). MACEs included cardiac death, TVR, non-TVR, rehospitalization due to UAP, and non-fatal MI. *P*<0.05 was considered statistically significant and was indicated in bold.

The median follow-up time was 36 months and impressive 99.0% of patients were successfully retained. Cumulative incidence plots showed that the incidence of MACEs in the FCR layer was lower than that in FIR layer (12.6% vs 24.0%, log-rank *P*<0.001) (**S3A Fig**). In the nonDM cohort, FCR layer (nonDM+FCR) also had a lower incidence of MACEs than FIR layer (nonDM+FIR) (9.8% vs 18.5%, log-rank *P* = 0.032) (**Fig 2B**). Similar situations also occurred in the DM cohort (16.1% vs 27.9%, log-rank *P* = 0.017) (**Fig 2C**).

**Fig 3:**
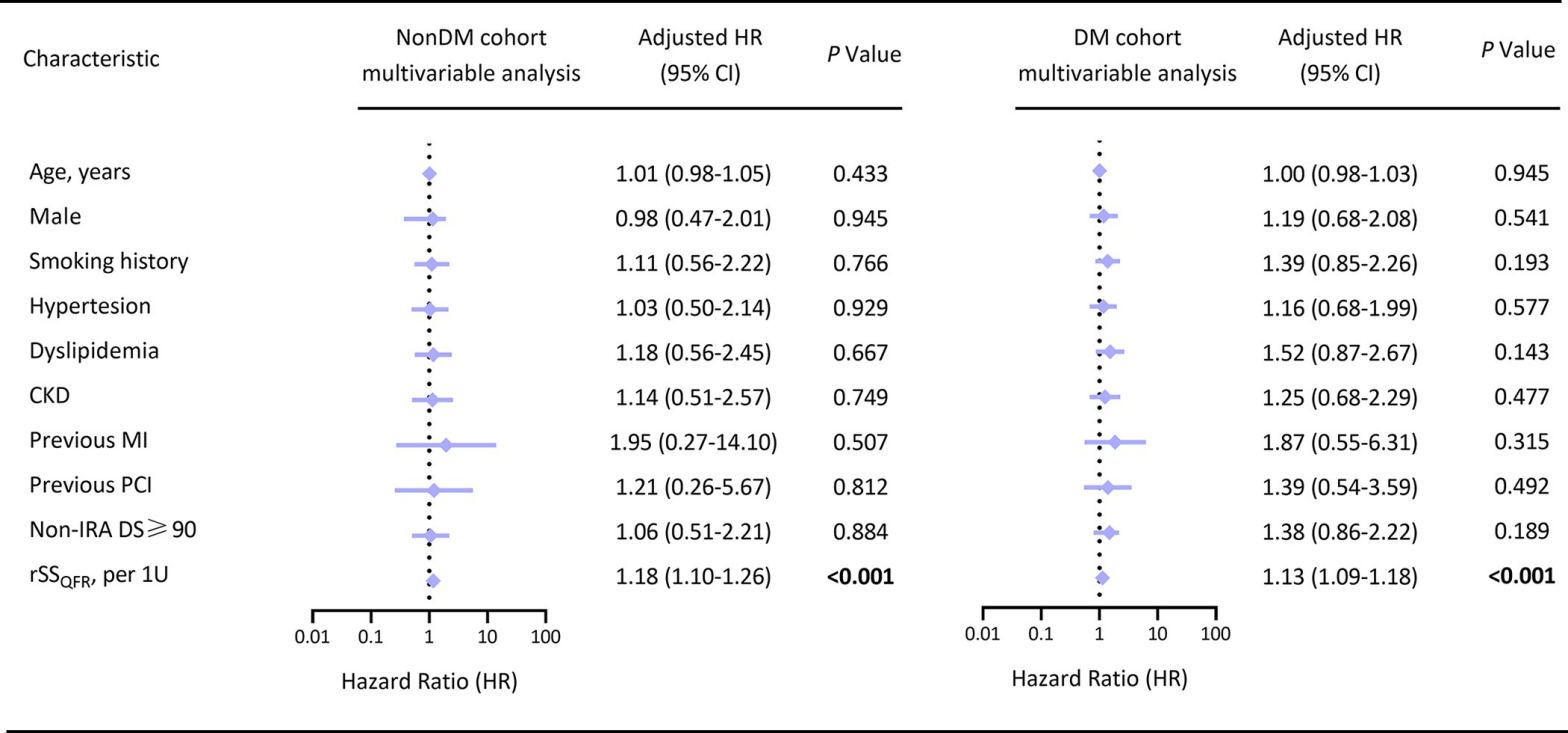
COX regression analysis for 3-year clinical outcomes. Multivariate Cox regression analysis for determining the relationship between rSS_QFR_ and MACEs in the nonDM cohort and DM cohort. Baseline characteristics including age, male, smoking history, hypertension, dyslipidemia, CKD, previous MI, previous PCI, and non-IRA DS≥90 were used to adjust the relationship. *P*<0.05 was considered statistically significant and was indicated in bold. Significance level alpha = 0.05 (95% CI).

Multivariate Cox regression revealed there was an association of rSS_QFR_ with MACEs that was independent of baseline characteristic differences in both nonDM cohort (HR (95% CI) = 1.18 (1.10–1.26), *P*<0.001) and DM cohort (HR (95% CI) = 1.13 (1.09–1.18), *P*<0.001) (**Fig 3**). Same situations occurred in overall patients (**S4 Fig**).

**Fig 4:**
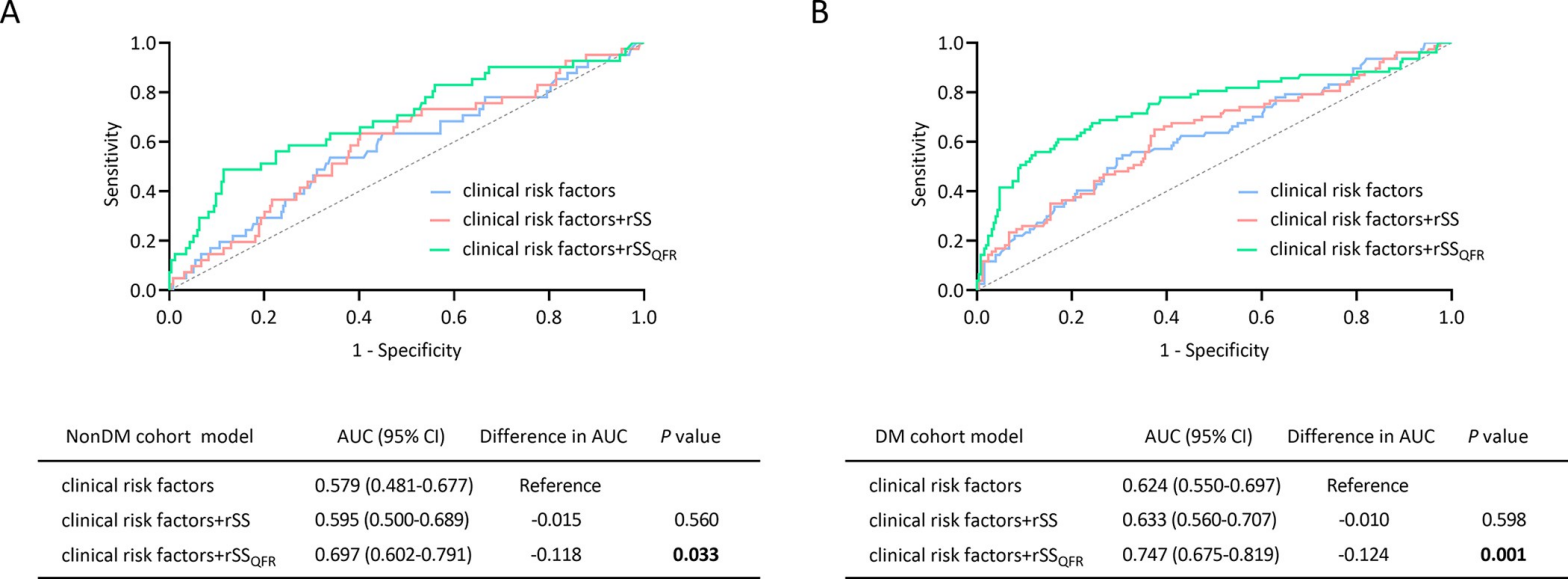
Prediction of 3-year clinical outcomes. ROC curve for predicting MACEs through rSS and rSS_QFR_ models in the nonDM cohort (**A**) and DM cohort (**B**). Clinical risk factors included age, male, smoking history, hypertension, dyslipidemia, CKD, previous MI, and previous PCI. *P*<0.05 was considered statistically significant and was indicated in bold.

ROC analysis showed adding rSS_QFR_ into the model of clinical risk factors yielded a significant improvement in prediction of MACEs, especially in the DM cohort (AUC (95% CI) = 0.747 (0.675–0.819), *P* = 0.001) than in the nonDM cohort (AUC (95% CI) = 0.697 (0.602–0.791), *P* = 0.033) (**Fig 4**). The rSS_QFR_ model was also applicable in overall patients (**S5 Fig**).

### Moderate lesions

**Fig 5:**
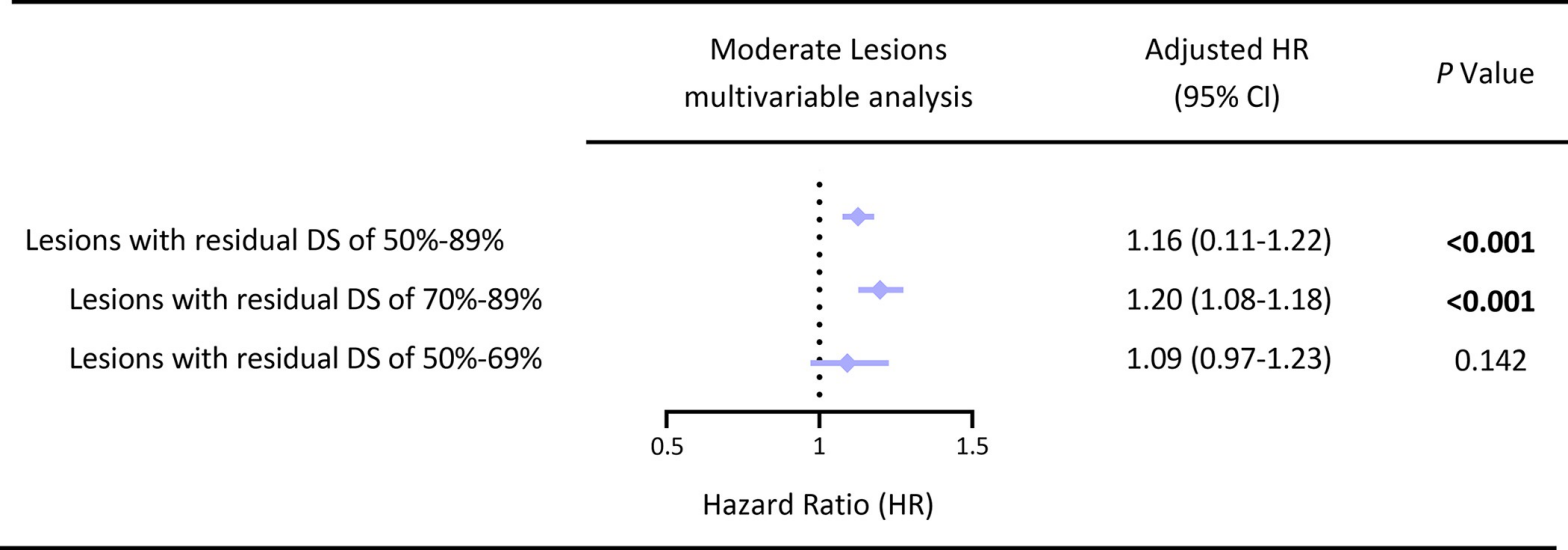
COX regression analysis for moderate lesions requiring revascularization. Multivariate Cox regression analysis of several subsets of moderate lesions between the rSS_QFR_ (per, 1U) and MACEs. Moderate lesions referred to lesions with residual DS of 50%-89%. *P*<0.05 was considered statistically significant and was indicated in bold. Significance level alpha = 0.05 (95% CI).

Furthermore, additional Cox analysis showed that rSS_QFR_ was also associated with an increased risk of MACEs in patients with moderate lesions (lesions with residual DS of 50%-89%) after procedure. Among them, the differences were observed in lesions with residual DS of 70%-89% (HR (95% CI) = 1.20 (1.08–1.18), *P*<0.001), rather than lesions with residual DS of 50%-69% (*P*>0.05) (**Fig 5**) (**S6 and S7 Figs**).

## Discussion

The major findings obtained from the study are as follows: 1) In patients with MVD-STEMI, the incidence of MACEs in FCR was lower than that in FIR, and the decrease was particularly significant in the DM cohort; 2) The association between QFR-derived rSS_QFR_ and MACEs was independent of baseline characteristic differences, and rSS_QFR_ provided higher prognostic predictive ability in DM cohort than in nonDM cohort; 3) QFR had the additional utility of identifying moderate residual lesions that require revascularization.

Previous studies showed that CR strategy could improve the outcomes of patients with MVD-STEMI [16]. Gustavo et al. reported that in MVD-STEMI patients, the incidence of MACEs was lower in the CR group than in the IR group [16]. However, it is controversial whether DM status affects the outcomes. For example, Kongyong proposed the CR benefits of MVD-STEMI patients still exist in nonDM, but disappear in DM [5]. In the COMPLETE trial, among MVD-STEMI patients the benefit of CR over culprit-lesion-only PCI (IR) was consistent regardless of the presence or absence of DM [6]. Thus, a controversy has arisen. Our study used QFR as a new cut-off point and proposed that the incidence of MACEs in FCR was lower than that in FIR in MVD-STEMI patients. In addition, we particularly found that the MACEs decrease from FIR to FCR in the DM cohort was more significant than that in the nonDM cohort (57.7% decrease vs 53.0% decrease). Although our study was retrospective, it suggested that FCR strategy may have greater benefits for the DM status of MVD-STEMI patients compared to nonDM status. Using the new tool, we explored the relationship between DM status and the benefits of different revascularization strategies of MVD-STEMI from a new perspective.

As for the reason why our MACEs were different from the previous literature, we considered for two points. Firstly, DM impairs the function of both macrovascular and microvascular coronary arteries [17]. CR evaluated by angiography in previous literature was based on anatomy and corresponded to macrovessels, while FCR evaluated by QFR in our study was based on functionality and corresponded to both macrovessels and microvessels. Undoubtedly, QFR-based FCR was closer to the true situation of coronary ischemia, especially in diseases with severe microcirculation dysfunction such as DM. This was also the mechanism why FCR could help reduce adverse events in DM patients. Secondly, different studies have different components of MACEs, may leading to differences in prognosis. The MACEs combination components used in this study followed the previous concepts of our research group [14]. Most components of MACEs in our study, such as cardiac death and TVR, were not significant, and significant differences in MACEs were mainly caused by rehospitalization due to UAP. This was because the study was a single-center study. And the study area is a vast cold area with a high incidence rate of heart disease. Therefore, people were more concerned about UAP and have a higher hospitalization rate in local hospitals due to UAP, especially after suffering from STEMI. As a result, the significant MACE estimates were mostly driven by rehospitalization due to UAP. Another reason was that this study was a small-sample study. The small sample size resulted in lack of differences in some single items. Future research will increase the number of research centers and expand the sample size to reduce this bias.

As for the reason why our non-IRA PCI ratio was relatively low, we also considered for two points. The first reason was that patients were affected by Diagnosis Related Groups (DRG) medical insurance payment and were almost unable to complete all IRA and non-IRA PCI procedures at once during the primary PCI period. Another reason was that the research area is a vast cold region, leading many patients to choose local hospitals for rehospitalization. However, many local hospitals lack the conditions or experience for intervention, resulting in fewer staged PCI procedures. Those resulted in a certain gap between the actual proportion of staged PCI and the theoretical proportion of staged PCI required for residual stenosis.

RSS_QFR_ integrates residual coronary artery severity and QFR values, serving as quantitative indicators for QFR-based FCR/FIR. To further investigate the impact of DM and rSS_QFR_ on MACEs risk, COX regression models are commonly used. In the FAVOR III China trial, FCR (rSS_QFR_ = 0) have been confirmed to be an independent predictor of MACEs in patients with CAD [13]. Our results indicated that the association between rSS_QFR_ (per 1U) and MACEs was independent of baseline characteristic differences in MVD-STEMI patients, whether combined with DM or nonDM. Our results were consistent with the direction of previous literature and may supplement data in the more severe CAD (MVD-STEMI) field.

Jiani et al. reported that the rSS_QFR_ model had higher predictive accuracy for MACEs in STEMI patients than the rSS model [10]. However, the prognostic value in MVD-STEMI patients remains unclear. Our research showed that rSS_QFR_ still had the ability to predict the prognosis of MVD-STEMI patients, and its ability was higher in the DM cohort than in the nonDM cohort. Therefore, our observations suggested that the application of the QFR test was more helpful in predicting prognosis and may be more beneficial to people with DM than those without DM.

What kind of moderate lesions (lesions with residual DS of 50%-89%) [18] require revascularization has always been a hot topic of clinical concern. John et al showed FFR assessment of moderate lesions can effectively guide revascularization in patients undergoing CABG and reduced the incidence rate of MACEs [19]. We found that in MVD-STEMI patients, lesions with residual DS of 70%-89% were more likely to require revascularization, while lesions with residual DS of 50%-69% may not require revascularization, as measured by the QFR test. Our research results fill the gap in QFR identification of moderate stenosis.

In conclusion, our study highlighted the use value of QFR in MVD-STEMI patients, especially those with DM. For centers equipped with QFR computation capabilities, our data supported the expanded use of this technique to optimize risk stratification and confirm the completeness of revascularization. Future randomized controlled studies comparing QFR to FFR in MVD-STEMI patients with DM are warranted to further explore the advantages of QFR.

### Study limitations

There are some potential limitations and biases in the design of this study. Firstly, being a single-center, retrospective, and observational study, our research was constrained by imbalances in baseline characteristics and selection bias. Secondly, the achievement of FCR or FIR depended on angiography video reviews, which were influenced by the preferences of interventional cardiologists/patients. Thirdly, the exclusion criteria introduced certain biases. This may have skewed the final results, and thus, the conclusions cannot be generalized to the excluded patient population. Fourthly, we did not consistently monitor blood glucose levels and drug compliance among DM patients throughout the study period. Consequently, it remains uncertain whether poor blood glucose management could exacerbate clinical outcomes. Fifthly, choosing cardiac death as the endpoint ignored the competitive risk of non-cardiac death, may affect the risk of cardiac death outcomes. Lastly, the study population was relatively small, making it challenging to detect differences in rare or low-frequency events.

## Conclusions

In patients with MVD-STEMI, the incidence of MACEs in FCR was lower than that in FIR, and the decrease was particularly significant in the DM cohort. The association between QFR-derived rSS_QFR_ and MACEs was independent of baseline characteristic differences, and rSS_QFR_ provided higher prognostic predictive ability in DM cohort than in nonDM cohort. Additionally, QFR had the additional utility of identifying moderate residual lesions that require revascularization.

## Supporting information

S1 File Definitions. (DOCX)

S1 Table Clinical characteristics between cohorts or layers. (DOCX)

S2 Table Procedural characteristics between cohorts or layers. (DOCX)

S3 Table 3-Year MACEs components between cohorts or layers. (DOCX)

S4 Table 3-Year MACEs components in cohorts. (DOCX)

S5 Table 3-Year MACEs components in layers. (DOCX)

S6 Table Data. (XLSX)

S1 Fig Distribution of rSS and rSS_QFR_. (DOCX)

S2 Fig Independent predictors of FIR. (DOCX)

S3 Fig Cumulative incidence plots for 3-year clinical outcomes in layers. (DOCX)

S4 Fig COX regression analysis for 3-year clinical outcomes in overall patients. (DOCX)

S5 Fig Prediction of 3-year clinical outcomes in overall patients. (DOCX)

S6 Fig COX regression analysis for moderate lesions requiring revascularization. (DOCX)

S7 Fig COX regression analysis for different moderate lesions requiring revascularization. (DOCX)

S1 Graphical abstract (TIF)
