# Supplementary material for: Association between quantitative flow ratio and clinical outcomes in multivessel disease STEMI patients with diabetes mellitus
Source: PLoS One. 2024 Dec 5;19(12):e0313892. doi: 10.1371/journal.pone.0313892 (PMC11620408; doi:10.1371/journal.pone.0313892)
Supplement: S1 File — (DOCX) [file pone.0313892.s001.docx]

**Methods**

**Definitions**

ST-elevation myocardial infarction (STEMI) was defined as persistent chest pain that lasted at least 30 minutes, arrival at the hospital within 12 hours from symptom onset, 12-lead electrocardiogram changes (ST-segment elevation of >0.1 mV in continuous leads or new-onset left bundle branch block), and elevation of cardiac biomarkers (creatine kinase-MB or cardiac troponin I) [1].

Diabetes mellitus (DM) was identified by a combination of strategies including patients reporting a history of DM, hemoglobin A1c (HbA1c) ≥6.5%, , and/or fasting blood glucose (FBG) ≥126 mg/dL, and/or the use of DM medication [2-5].

Hypertension was defined as systolic pressure of 140 mm Hg or greater, diastolic pressure of 90 mm Hg or greater, or use of antihypertensive medications [6].

Chronic kidney disease (CKD) was defined as an estimated glomerular filtration rate (eGFR) less than 60 mL/min/1.73 m^2^ on at least 2 consecutive visits, microalbuminuria (albumin excretion rate 30 mg/per 24 hours on 2 consecutive visits), or macroalbuminuria (albumin excretion rate 300 mg/per 24 hours) [7].

Multivessel disease (MVD) was defined at least one non-infarct-related artery (non-IRA) with ≥50% lumen diameter stenosis. Single-vessel disease was not considered because it was definitely complete revascularization (CR) without randomization.

Cardiac death referred to any death caused by myocardial infarction, cardiac perforation or pericardial tamponade, arrhythmia or conduction abnormalities, surgical complications, or inability to rule out cardiac causes [1].

Ischemia-driven revascularization was defined as repeated percutaneous or surgical revascularization of target vessel or non-target vessel identified in the index procedure, accompanied by ischemic symptoms. For lesions identified in index coronary angiography, planned revascularization was not considered an adverse event [1].

Unstable angina pectoris (UAP) were evaluated according to Braunwald Unstable Angina Classification [1].

Non-fatal myocardial infarction (MI) was based on the detection of the rise and fall of cardiac biomarkers (preferably troponin) above the 99th percentile of the reference upper limit, as well as evidence of myocardial ischemia, meeting at least one of the following criteria: ischemic symptoms; changes in electrocardiogram indicate new ischemia (new ST-T changes or new left bundle branch block); the development of pathological Q waves in electrocardiogram; and imaging evidence of new surviving myocardial loss or new local wall motion abnormalities [1].

**References**

| [1] | Fang C, Yin Y, Jiang S, Zhang S, Wang J, Wang Y, et al. Increased Vulnerability and Distinct Layered Phenotype at Culprit and Nonculprit Lesions in STEMI Versus NSTEMI. JACC Cardiovasc Imaging. 2022;15(4):672-681. doi: 10.1016/j.jcmg.2021.07.022. |
| --- | --- |
| [2] | Hamid A, Yimer WK, Oshunbade AA, Kamimura D, Clark D 3rd, Fox ER, et al. Impact of Diabetes and Hypertension on Left Ventricular Structure and Function: The Jackson Heart Study. J Am Heart Assoc. 2023;12(6):e026463. doi: 10.1161/JAHA.122.026463. |
| [3] | Wang L, Li X, Wang Z, Bancks MP, Carnethon MR, Greenland P, et al. Trends in Prevalence of Diabetes and Control of Risk Factors in Diabetes Among US Adults, 1999-2018. JAMA. 2021;326(8):1–13. doi: 10.1001/jama.2021.9883. |
| [4] | Li N, Zhou J, Chen R, Zhao X, Li J, Zhou P, et al. Prognostic impacts of diabetes status and lipoprotein(a) levels in patients with ST-segment elevation myocardial infarction: a prospective cohort study. Cardiovasc Diabetol. 2023;22(1):151. doi: 10.1186/s12933-023-01881-w. |
| [5] | Song L, Zhao X, Chen R, Li J, Zhou J, Liu C, et al. Association of PCSK9 with inflammation and platelet activation markers and recurrent cardiovascular risks in STEMI patients undergoing primary PCI with or without diabetes. Cardiovasc Diabetol. 2022;21(1):80. doi: 10.1186/s12933-022-01519-3. |
| [6] | Castañer O, Pintó X, Subirana I, Amor AJ, Ros E, Hernáez Á, et al. Remnant Cholesterol, Not LDL Cholesterol, Is Associated With Incident Cardiovascular Disease. J Am Coll Cardiol. 2020;76(23):2712-2724. doi: 10.1016/j.jacc.2020.10.008. |
| [7] | Braffett BH, Bebu I, El Ghormli L, Cowie CC, Sivitz WI, Pop-Busui R, et al. Cardiometabolic Risk Factors and Incident Cardiovascular Disease Events in Women vs Men With Type 1 Diabetes. JAMA Netw Open. 2022;5(9):e2230710. doi: 10.1001/jamanetworkopen.2022.30710. |
