# Supplementary material for: Association between quantitative flow ratio and clinical outcomes in multivessel disease STEMI patients with diabetes mellitus
Source: PLoS One. 2024 Dec 5;19(12):e0313892. doi: 10.1371/journal.pone.0313892 (PMC11620408; doi:10.1371/journal.pone.0313892)
Supplement: S1 Table — (DOCX) [file pone.0313892.s002.docx]

**S1 Table. Clinical Characteristics Between Cohorts or Layers.**

|  | **Cohorts** | | |  | **Layers** | | |
| --- | --- | --- | --- | --- | --- | --- | --- |
|  | **NonDM cohort (n=295)** | **DM cohort**  **(n=328)** | ***P* Value** |  | **FCR layer**  **(n=302)** | **FIR layer**  **(n=321)** | ***P* Value** |
| Age, y | 60.5 ± 11.3 | 61.3 ± 10.3 | 0.363 |  | 60.6 ± 10.8 | 61.3 ± 10.7 | 0.444 |
| Male | 190 (64.4) | 209 (63.7) | 0.858 |  | 195 (64.6) | 204 (63.6) | 0.791 |
| Coronary risk factors |  |  |  |  |  |  |  |
| Current smoking | 169 (57.3) | 158 (48.2) | **0.023** |  | 163 (54.0) | 164 (51.1) | 0.471 |
| Hypertension | 205 (69.5) | 232 (70.7) | 0.736 |  | 197 (65.2) | 240 (74.8) | **0.009** |
| Dyslipidemia | 222 (75.3) | 237 (72.3) | 0.396 |  | 227 (75.2) | 232 (72.3) | 0.413 |
| CKD | 75 (25.4) | 82 (25.0) | 0.903 |  | 74 (24.5) | 83 (25.9) | 0.697 |
| Previous history |  |  |  |  |  |  |  |
| Previous MI | 4 (1.4) | 10 (3.0) | 0.155 |  | 6 (2.0) | 8 (2.5) | 0.671 |
| Previous PCI | 8 (2.7) | 11 (3.4) | 0.642 |  | 9 (3.0) | 10 (3.1) | 0.922 |
| Laboratory data |  |  |  |  |  |  |  |
| TC, mg/dl | 4.7 (4.1-5.6) | 5.0 (4.2-5.7) | 0.109 |  | 4.8 (4.1-5.6) | 5.0 (4.2-5.8) | 0.064 |
| TG, mg/dL | 1.6 (1.1-2.0) | 1.4 (0.9-2.1) | 0.123 |  | 1.5 (1.0-1.9) | 1.5 (1.0-2.2) | 0.138 |
| LDL-C, mg/dL | 2.8 (2.3-3.5) | 3.1 (2.5-3.7) | **<0.001** |  | 2.8 (2.4-3.5) | 3.1 (2.4-3.7) | 0.078 |
| HDL-C, mg/dL | 1.2 (1.0-1.4) | 1.3 (1.1-1.5) | **0.002** |  | 1.2 (1.1-1.5) | 1.3 (1.1-1.5) | 0.517 |
| Peak cTnI, μg/L | 59.8 (26.5-106.6) | 81.1 (32.3-152.8) | **0.003** |  | 61.4 (25.6-127.9) | 75.2 (29.4-137.3) | 0.137 |
| LVEF, % | 61.0 (55.0-62.0) | 60.0 (53.0-62.0) | 0.182 |  | 61.0 (55.0-63.0) | 60.0 (54.0-62.0) | 0.313 |
| eGFR, mL/min/1.73 m^2^ | 75.5 (59.6-95.7) | 73.8 (60.0-90.5) | 0.284 |  | 77.4 (60.3-94.7) | 73.1 (59.9-90.5) | 0.172 |
| HbA1c, % | 5.6 (5.4-5.8) | 6.5 (5.7-8.0) | **<0.001** |  | 5.8 (5.5-6.3) | 5.9 (5.5-7.1) | **0.024** |
| FBG, mmol/L | 5.4 (4.9-6.2) | 8.9 (7.5-11.8) | **<0.001** |  | 6.6 (5.2-8.3) | 7.4 (5.6-9.9) | **<0.001** |
| Diabetes management |  |  |  |  |  |  |  |
| Diet or exercise | - | 15 (4.6%) | - |  | 4 (2.9) | 11 (25.8) | 0.216 |
| Oral agent | - | 192 (58.5%) | - |  | 82 (59.4) | 110 (57.9) | 0.782 |
| Injection | - | 51 (15.5%) | - |  | 14 (10.1) | 37 (19.5) | **0.021** |
| Untreated | - | 91 (27.7%) | - |  | 42 (30.4) | 49 (25.8) | 0.354 |
| Discharge medications |  |  |  |  |  |  |  |
| Aspirin | 291 (98.6) | 321 (97.9) | 0.461 |  | 296 (98.0) | 316 (98.4) | 0.684 |
| P2Y12 inhibitors | 292 (99.0) | 326 (99.4) | 0.570 |  | 299 (99.0) | 319 (99.4) | 0.605 |
| Statins | 289 (98.0) | 326 (99.4) | 0.115 |  | 299 (99.0) | 316 (98.4) | 0.532 |
| Oral anticoagulant | 32 (10.8) | 37 (11.3) | 0.863 |  | 33 (10.9) | 36 (11.2) | 0.909 |

Values are n (%), mean±SD, or median (interquartile range). Bold represented significance between nonDM cohort and DM cohort or between FCR layer and FIR layer. *P*<0.05 was considered statistically significant.
