# Supplementary material for: Association between quantitative flow ratio and clinical outcomes in multivessel disease STEMI patients with diabetes mellitus
Source: PLoS One. 2024 Dec 5;19(12):e0313892. doi: 10.1371/journal.pone.0313892 (PMC11620408; doi:10.1371/journal.pone.0313892)
Supplement: S2 Table — (DOCX) [file pone.0313892.s003.docx]

**S2 Table. Procedural Characteristics Between Cohorts or Layers.**

|  | **Cohorts** | | |  | **Layers** | | |
| --- | --- | --- | --- | --- | --- | --- | --- |
|  | **NonDM cohort (n=295)** | **DM cohort**  **(n=328)** | ***P* Value** |  | **FCR layer**  **(n=302)** | **FIR layer**  **(n=321)** | ***P* Value** |
| 3-vessel diseases | 157 (53.2%) | 193 (58.8%) | 0.158 |  | 140 (46.4%) | 210 (65.4%) | **<0.001** |
| Syntax score |  |  |  |  |  |  |  |
| Angiography-derived |  |  |  |  |  |  |  |
| Pre-PCI (SS) | 17.0 (13.0-21.5) | 18.0 (14.5-23.0) | **0.014** |  | 15.0 (12.0-19.5) | 16.3 (13.0-21.0) | **0.007** |
| Post-PCI (rSS) | 6.0 (3.0-9.0) | 7.0 (5.0-11.0) | **0.002** |  | 5.0 (2.0-8.0) | 9.0 (6.0-12.0) | **<0.001** |
| QFR-derived |  |  |  |  |  |  |  |
| Pre-PCI (SS_QFR_) | 8.0 (6.0-15.5) | 12.0 (6.0-18.5) | **<0.001** |  | 6.0 (4.5-13.5) | 13.0 (8.5-19.3) | **<0.001** |
| Post-PCI (rSS_QFR_) | 0 (0-2.0) | 2.0 (0-6.0) | **<0.001** |  | 0 | 4.0 (2.0-8.0) | **<0.001** |
| IRA |  |  |  |  |  |  |  |
| Number | 1 | 1 | - |  | 1 | 1 | - |
| Initial TIMI flow grade ≤ 1 | 292 (99.0%) | 318 (97.0%) | 0.076 |  | 299 (99.0%) | 311 (96.9%) | 0.064 |
| DS ≥ 90 | 294 (99.7%) | 320 (97.6%) | **0.028** |  | 300 (99.3%) | 314 (97.8%) | 0.112 |
| QFR ≤ 0.8 | 269 (91.2%) | 308 (93.9%) | 0.196 |  | 274 (90.7%) | 303 (94.4%) | 0.080 |
| Location |  |  |  |  |  |  |  |
| LAD | 122 (41.4%) | 137 (41.8%) | 0.917 |  | 123 (40.7%) | 136 (42.4%) | 0.678 |
| LCX | 39 (13.2%) | 46 (14.0%) | 0.770 |  | 36 (11.9%) | 49 (15.3%) | 0.224 |
| RCA | 134 (45.4%) | 145 (44.2%) | 0.760 |  | 143 (47.4%) | 136 (42.4%) | 0.211 |
| Location of QFR ≤ 0.8 |  |  |  |  |  |  |  |
| LAD | 111 (37.6%) | 130 (39.6%) | 0.608 |  | 115 (38.1%) | 126 (39.3%) | 0.764 |
| LCX | 39 (13.2%) | 41 (12.5%) | 0.788 |  | 33 (10.9%) | 47 (14.6%) | 0.166 |
| RCA | 119 (40.3%) | 137 (41.8%) | 0.717 |  | 126 (41.7%) | 130 (40.5%) | 0.756 |
| Non-IRA |  |  |  |  |  |  |  |
| Number |  |  |  |  |  |  |  |
| Pre-PCI | 2.0 (1.0-2.0) | 2.0 (1.0-2.0) | **<0.001** |  | 1.0 (1.0-2.0) | 2.0 (1.0-2.0) | **<0.001** |
| Post-PCI | 1.0 (1.0-2.0) | 2.0 (1.0-2.0) | **0.003** |  | 1.0 (1.0-2.0) | 2.0 (1.0-2.0) | **<0.001** |
| DS ≥ 90 |  |  |  |  |  |  |  |
| Pre-PCI | 78 (26.4%) | 109 (33.2%) | 0.065 |  | 40 (13.2%) | 147 (45.8%) | **<0.001** |
| Post-PCI | 61 (20.7%) | 94 (28.7%) | **0.021** |  | 23 (7.6%) | 132 (41.1%) | **<0.001** |
| QFR ≤ 0.8 |  |  |  |  |  |  |  |
| Pre-PCI | 129 (43.7%) | 194 (59.1%) | **<0.001** |  | 21 (7.0%) | 302 (94.1%) | **<0.001** |
| Post-PCI | 118 (40.0%) | 179 (54.6%) | **<0.001** |  | 0 | 297 (92.5%) | **<0.001** |
| Location |  |  |  |  |  |  |  |
| LAD | 156 (52.9%) | 178 (54.3%) | 0.729 |  | 156 (51.7%) | 178 (55.5%) | 0.342 |
| LCX | 176 (59.7%) | 210 (64.0%) | 0.263 |  | 167 (55.3%) | 219 (68.2%) | **<0.001** |
| RCA | 119 (40.3%) | 156 (47.6%) | 0.070 |  | 118 (39.1%) | 157 (48.9%) | **0.013** |
| Location of QFR ≤ 0.8 |  |  |  |  |  |  |  |
| LAD | 53 (18.0%) | 75 (22.9%) | 0.131 |  | 11 (3.6%) | 117 (36.4%) | **<0.001** |
| LCX | 67 (22.7%) | 104 (31.7%) | **0.012** |  | 7 (2.3%) | 164 (51.1%) | **<0.001** |
| RCA | 39 (13.2%) | 81 (24.7%) | **<0.001** |  | 4 (1.3%) | 116 (36.1%) | **<0.001** |
| Treatment |  |  |  |  |  |  |  |
| Number of stents | 1.0 (1.0-1.0) | 1.0 (1.0-2.0) | **0.008** |  | 1.0 (1.0-1.0) | 1.0 (1.0-2.0) | 0.076 |
| Stent diameter, mm | 3.0 (2.8-3.5) | 3.0 (2.8-3.3) | 0.064 |  | 3.0 (2.8-3.5) | 3.0 (2.8-3.3) | **0.003** |
| Stent length, mm | 29.0 (23.0-36.0) | 30.0 (23.3-46.0) | 0.063 |  | 29.0 (23.0-38.0) | 33.0 (24.0-44.0) | **0.006** |
| Thrombectomy | 223 (75.6%) | 234 (71.3%) | 0.231 |  | 230 (76.2%) | 227 (70.7%) | 0.125 |
| IRA PCI | 100% | 100% | - |  | 100% | 100% | - |
| Non-IRA PCI | 32 (10.8%) | 36 (11.0%) | 0.959 |  | 29 (9.6%) | 39 (12.1%) | 0.308 |
| Primary PCI | 17 (5.8%) | 18 (5.5%) | 0.882 |  | 24 (7.9%) | 11 (3.4%) | **0.014** |
| Staged PCI | 15 (5.1%) | 18 (5.5%) | 0.823 |  | 5 (1.7%) | 28 (8.7%) | **<0.001** |

Values are n (%), mean±SD, or median (interquartile range). Bold represented significance between nonDM cohort and DM cohort or between FCR layer and FIR layer. *P*<0.05 was considered statistically significant.
