# Supplementary material for: Association between quantitative flow ratio and clinical outcomes in multivessel disease STEMI patients with diabetes mellitus
Source: PLoS One. 2024 Dec 5;19(12):e0313892. doi: 10.1371/journal.pone.0313892 (PMC11620408; doi:10.1371/journal.pone.0313892)
Supplement: S3 Table — (DOCX) [file pone.0313892.s004.docx]

**S3 Table. 3-Year MACEs Components Between Cohorts or Layers.**

|  | **Cohorts** | | | | |  | **Layers** | | | |
| --- | --- | --- | --- | --- | --- | --- | --- | --- | --- | --- |
|  | **NonDM cohort**  **(n=295)** | **DM cohort**  **(n=328)** | **OR (95%CI)** | ***P* value** | |  | **FCR Layer**  **(n=302)** | **FIR Layer**  **(n=321)** | **OR (95%CI)** | ***P* value** |
| Cardiac death | 0* | 1 (0.3)* | 1.81 (0.33-9.91) | | 0.492 |  | 0* | 1 (0.3)* | 1.89 (0.35-10.34) | 0.461 |
| TVR | 15 (5.1) | 13 (4.0) | 0.78 (0.37-1.64) | | 0.510 |  | 10 (3.3) | 18 (5.6) | 1.70 (0.79-3.69) | 0.176 |
| Non-TVR | 16 (5.4) | 14 (4.3) | 0.78 (0.38-1.60) | | 0.502 |  | 7 (2.3) | 23 (7.2) | 3.19 (1.37-7.44) | **0.007** |
| Rehospitalization due to UAP | 26 (8.8) | 62 (19.1) | 2.36 (1.49-3.72) | | **<0.001** |  | 33 (10.9) | 55 (17.3) | 1.65 (1.08-2.54) | **0.022** |
| Non-fatal MI | 2 (0.7) | 13 (4.0) | 6.00 (1.35-26.60) | | **0.018** |  | 3 (1.0) | 12 (3.8) | 3.82 (1.08-13.55) | **0.038** |

Values are n (%), mean±SD, or median (interquartile range). Bold represented significance between nonDM cohort and DM cohort or between FCR layer and FIR layer. MACEs components included cardiac death, TVR, non-TVR, rehospitalization due to UAP, and non-fatal MI. *P*<0.05 was considered statistically significant. *In cases where zero events were observed, both the experimental group and the control group added 0.5 events through data imputation techniques to facilitate subsequent analysis.
