# Supplementary material for: Association between quantitative flow ratio and clinical outcomes in multivessel disease STEMI patients with diabetes mellitus
Source: PLoS One. 2024 Dec 5;19(12):e0313892. doi: 10.1371/journal.pone.0313892 (PMC11620408; doi:10.1371/journal.pone.0313892)
Supplement: S4 Table — (DOCX) [file pone.0313892.s005.docx]

**S4 Table. 3-Year MACEs Components in Cohorts.**

|  | **NonDM cohort** | | | |  | **DM cohort** | | | |
| --- | --- | --- | --- | --- | --- | --- | --- | --- | --- |
|  | **NonDM+FCR**  **(n=164)** | **NonDM+FIR**  **(n=131)** | **OR (95%CI)** | ***P* value** |  | **DM+FCR**  **(n=138)** | **DM+FIR**  **(n=190)** | **OR (95%CI)** | ***P* value** |
| Cardiac death | 0 | 0 | - | - |  | 0* | 1 (0.5)* | 1.10 (0.18-6.56) | 0.920 |
| TVR | 6 (3.7) | 9 (6.9) | 1.87 (0.67-5.25) | 0.235 |  | 4 (2.9) | 9 (4.8) | 1.66 (0.51-5.38) | 0.400 |
| Non-TVR | 6 (3.7) | 10 (7.6) | 2.15 (0.78-5.92) | 0.138 |  | 1 (0.7) | 13 (6.9) | 9.86 (1.29-75.34) | **0.027** |
| Rehospitalization due to UAP | 13 (7.9) | 13 (9.9) | 1.26 (0.58-2.72) | 0.557 |  | 20 (14.5) | 42 (22.4) | 1.62 (0.96-2.76) | 0.073 |
| Non-fatal MI | 0* | 2 (1.5)* | 3.79 (0.39-36.46) | 0.248 |  | 0* | 10 (5.4)* | 2.04 (0.65-6.39) | 0.224 |

Values are n (%), mean±SD, or median (interquartile range). Bold represented significance in the nonDM cohort or in the DM cohort. MACEs components included cardiac death, TVR, non-TVR, rehospitalization due to UAP, and non-fatal MI. *P*<0.05 was considered statistically significant. *In cases where zero events were observed, both the experimental group and the control group added 0.5 events through data imputation techniques to facilitate subsequent analysis.
