# Supplementary material for: Association between quantitative flow ratio and clinical outcomes in multivessel disease STEMI patients with diabetes mellitus
Source: PLoS One. 2024 Dec 5;19(12):e0313892. doi: 10.1371/journal.pone.0313892 (PMC11620408; doi:10.1371/journal.pone.0313892)
Supplement: S5 Table — (DOCX) [file pone.0313892.s006.docx]

**S5 Table****. 3-Year MACEs Components in Layers.**

|  | **FCR layer** | | | | | | |  | **FIR layer** | | | |
| --- | --- | --- | --- | --- | --- | --- | --- | --- | --- | --- | --- | --- |
|  | **NonDM+FCR**  **(n=164)** | **DM+FCR**  **(n=138)** | | **OR (95%CI)** | | ***P* value** | |  | **NonDM+FIR**  **(n=131)** | **DM+FIR**  **(n=190)** | **OR (95%CI)** | ***P* value** |
| Cardiac death | 0 | | 0 | | - | | - |  | 0* | 1 (0.5)* | 1.05 (0.17-6.26) | 0.961 |
| TVR | 6 (3.7) | | 4 (2.9) | | 0.79 (0.22-2.79) | | 0.711 |  | 9 (6.9) | 9 (4.8) | 0.69 (0.27-1.74) | 0.434 |
| Non-TVR | 6 (3.7) | | 1 (0.7) | | 0.20 (0.02-1.63) | | 0.131 |  | 10 (7.6) | 13 (6.9) | 0.89 (0.39-2.03) | 0.778 |
| Rehospitalization due to UAP | 13 (7.9) | | 20 (14.5) | | 1.93 (0.96-3.88) | | 0.065 |  | 13 (9.9) | 42 (22.4) | 2.51 (1.35-4.67) | **0.004** |
| Non-fatal MI | 0* | | 3 (2.2)* | | 4.82 (0.54-43.16) | | 0.159 |  | 2 (1.5) | 10 (5.4) | 3.56 (0.78-16.23) | 0.101 |

Values are n (%), mean±SD, or median (interquartile range). Bold represented significance in the FCR layer or in the FIR layer. MACEs components included cardiac death, TVR, non-TVR, rehospitalization due to UAP, and non-fatal MI. *P*<0.05 was considered statistically significant. *In cases where zero events were observed, both the experimental group and the control group added 0.5 events through data imputation techniques to facilitate subsequent analysis.
