# Supplementary material for: Association between quantitative flow ratio and clinical outcomes in multivessel disease STEMI patients with diabetes mellitus
Source: PLoS One. 2024 Dec 5;19(12):e0313892. doi: 10.1371/journal.pone.0313892 (PMC11620408; doi:10.1371/journal.pone.0313892)

**S1 Fig. Distribution of rSS and rSS_QFR_.**

Violin plot for distribution of rSS (**A**) and rSS_QFR_ (**B**) in the four groups. The value of rSS_QFR_ was 0 in FCR-related groups, therefore it can not be displayed in the picture.


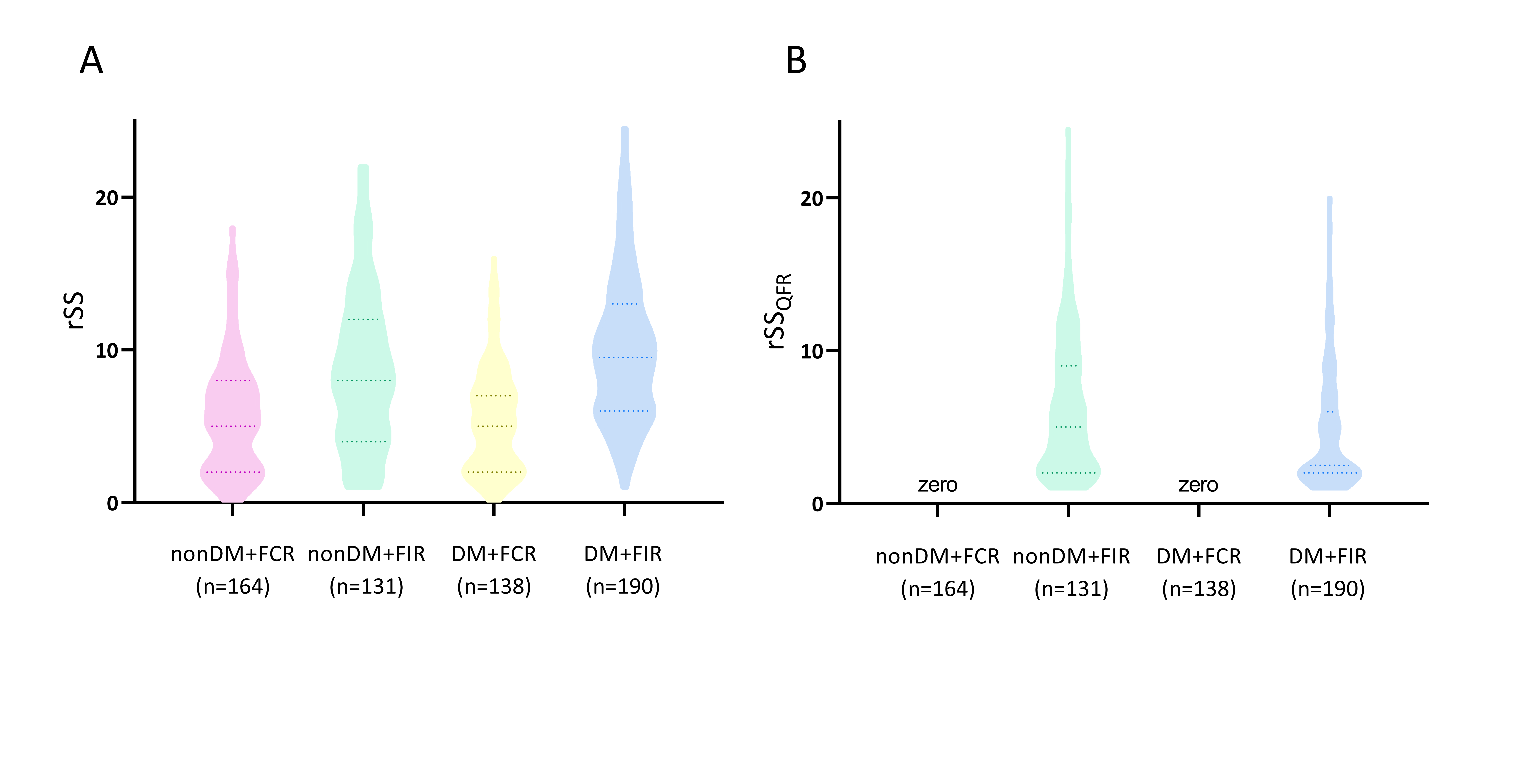

Supplement: S1 Fig — (DOCX) [file pone.0313892.s008.docx]
