# Supplementary material for: Association between quantitative flow ratio and clinical outcomes in multivessel disease STEMI patients with diabetes mellitus
Source: PLoS One. 2024 Dec 5;19(12):e0313892. doi: 10.1371/journal.pone.0313892 (PMC11620408; doi:10.1371/journal.pone.0313892)

**S2 Fig. Independent Predictors of FIR.**

Multivariate logistic regression analysis for independent predictors of FIR in the nonDM cohort and DM cohort. Baseline characteristics included age, male, smoking history, hypertension, dyslipidemia, CKD, previous MI, previous PCI, SS, SS_QFR_, and non-IRA DS ≥ 90. *P*<0.05 was considered statistically significant and was indicated in bold. Significance level alpha =0.05 (95% CI).


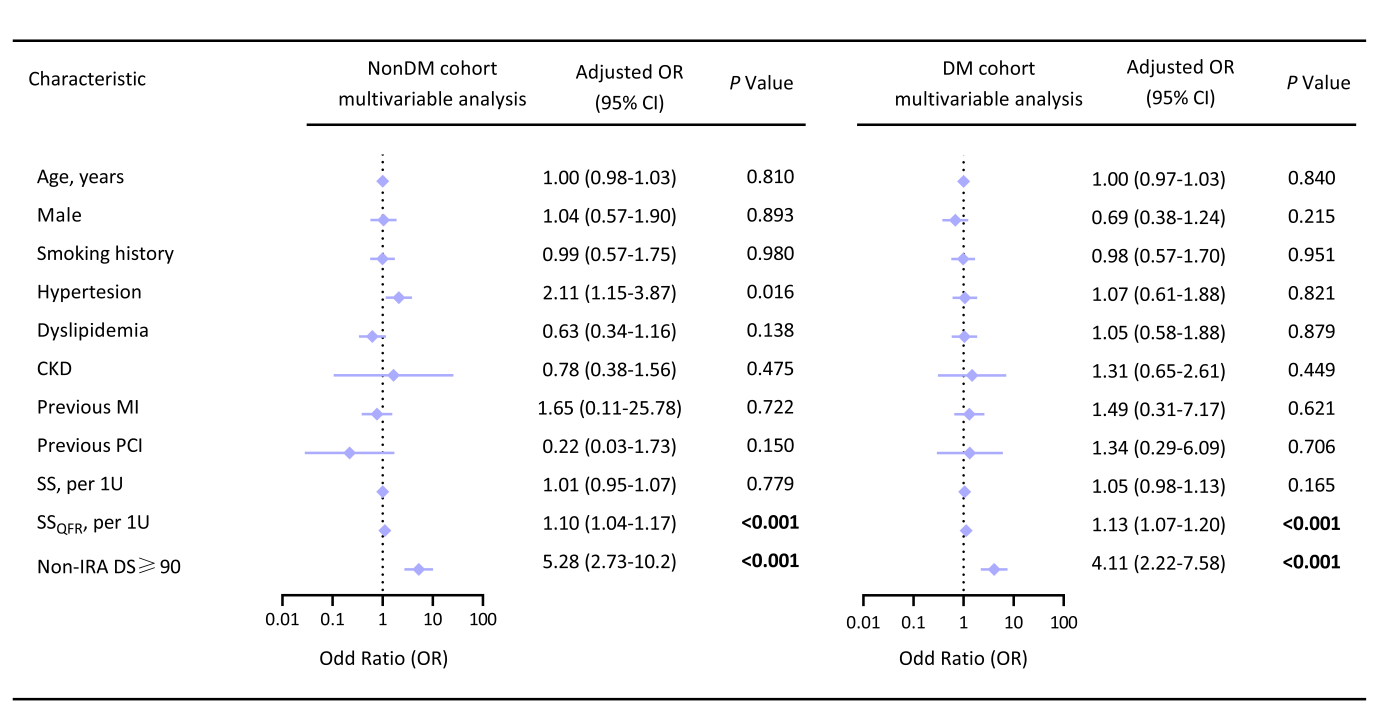

Supplement: S2 Fig — (DOCX) [file pone.0313892.s009.docx]
