# Supplementary material for: Association between quantitative flow ratio and clinical outcomes in multivessel disease STEMI patients with diabetes mellitus
Source: PLoS One. 2024 Dec 5;19(12):e0313892. doi: 10.1371/journal.pone.0313892 (PMC11620408; doi:10.1371/journal.pone.0313892)
Supplement: S3 Fig — (DOCX) [file pone.0313892.s010.docx]

**S3 Figure. Cumulative Incidence Plots for 3-Year Clinical Outcomes in Layers.**

MACEs plots and MACEs components histogram for comparison between FCR and FIR layers (**A**), comparison between nonDM and DM cohorts in the FCR layer (**B**), and comparison between nonDM and DM cohorts in the FIR layer (**C**). MACEs included cardiac death, TVR, non-TVR, rehospitalization due to UAP, and non-fatal MI. *P*<0.05 was considered statistically significant and was indicated in bold.

**

**
