# Supplementary material for: Association between quantitative flow ratio and clinical outcomes in multivessel disease STEMI patients with diabetes mellitus
Source: PLoS One. 2024 Dec 5;19(12):e0313892. doi: 10.1371/journal.pone.0313892 (PMC11620408; doi:10.1371/journal.pone.0313892)

**S5 Fig. Prediction of 3-year Clinical Outcomes in Overall Patients.**

ROC curve for predicting MACEs through rSS and rSS_QFR_ models in the overall patients. Clinical risk factors included age, male, smoking history, hypertension, dyslipidemia, CKD, previous MI, previous PCI. *P*<0.05 was considered statistically significant and was indicated in bold.


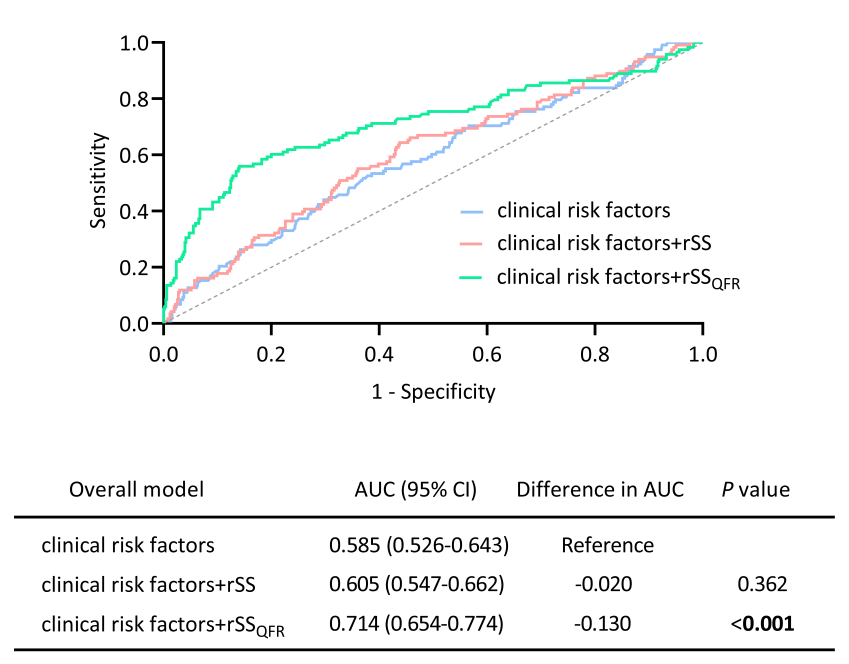

Supplement: S5 Fig — (DOCX) [file pone.0313892.s012.docx]
