# Supplementary material for: Association between quantitative flow ratio and clinical outcomes in multivessel disease STEMI patients with diabetes mellitus
Source: PLoS One. 2024 Dec 5;19(12):e0313892. doi: 10.1371/journal.pone.0313892 (PMC11620408; doi:10.1371/journal.pone.0313892)

**S6 Fig. COX Regression Analysis for Moderate Lesions Requiring Revascularization.**

Multivariate Cox regression analysis between the rSS_QFR_ (per, 1U) and MACEs in moderate lesions. Moderate lesions referred to lesions with residual DS of 50%-89%. Baseline characteristics included age, male, smoking history, hypertension, dyslipidemia, CKD, previous MI, previous PCI, non-IRA DS≥90, and DM. *P*<0.05 was considered statistically significant and was indicated in bold. Significance level alpha = 0.05 (95% CI).


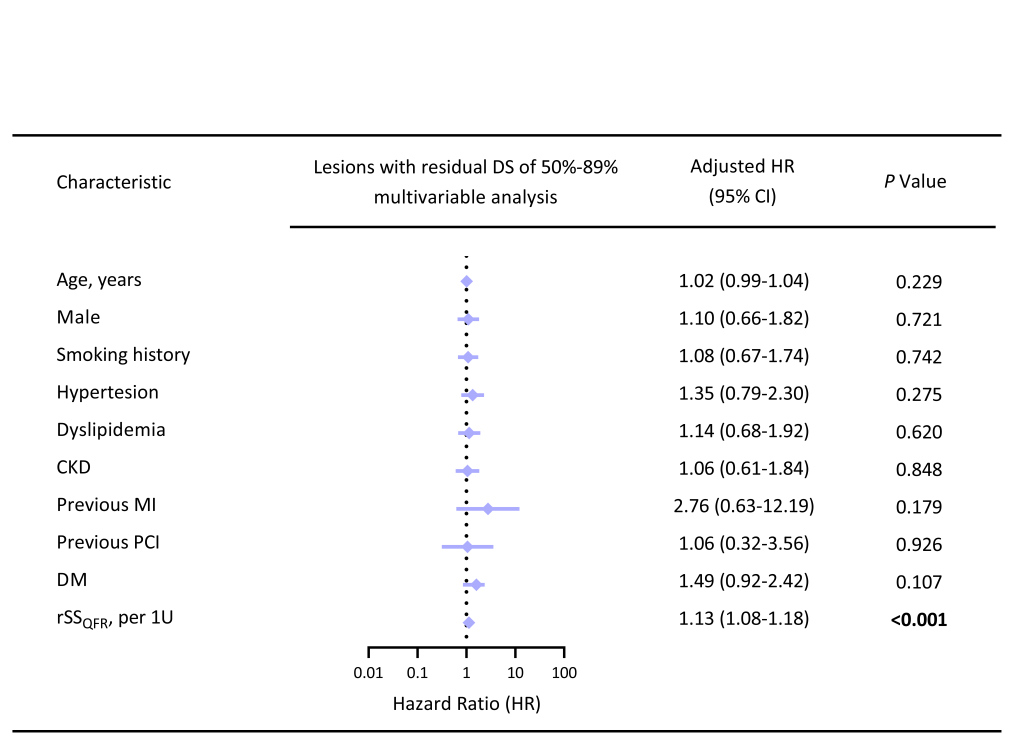

Supplement: S6 Fig — (DOCX) [file pone.0313892.s013.docx]
